# Glances at Foreign Asylums

**Published:** 1890-06-07

**Authors:** 


### Glances at Foreign Asylums.

[Annual Reports, Pamphlets, &c., should be addressed to The Editor, The Lodge, Porchester Square, London, W.]

### BUFFALO STATE ASYLUM.

At the close of the year this institution contained 1,368 patients. There were 274 admissions, 82 recoveries, and 45 deaths. The percentage of recoveries calculated in the English way stands at 29·92, which is by no means high; but we note that 22 cases were discharged as "much improved," which would considerably modify the percentage, as doubtless many of them ultimately recovered.

### BOSTON LUNATIC HOSPITAL.

Dr. Fisher presents us with a very clear and able report. The hospital contains about 180 beds, and it would seem to be almost always full or on the verge thereof; indeed, there was a daily average number of 173 resident. The admissions were 185; the discharges, 167; and the deaths, 41. The percentage of recoveries was decidedly low, standing at only 18, but it ought to be stated that this low rate is exceptional, and the average for the last seven years has been 33 per cent.

Dr. Fisher points out that the lack of single-bedded rooms is a serious defect in the construction of the asylum, and he attributes the increase of mechanical restraint and of chemical restraint to this defect. Our English training would lead us to doubt whether the defect in construction can thus be atoned for, or whether the defect can be put forward as an excuse for the restraint.

The weekly rate of maintenance is about £1 7*s.* 4*d.* This is an increase over the previous year, and is accounted for by a decrease in the numbers. The effect which numbers have on the rate is very clearly put as follows: "A hospital for the insane is like a publishing house, which must have a certain plant and organization to do business at all. It cannot sell the first or the second hundred impressions of a book as cheaply as its neighbour can sell a thousand."

### HOSPITAL FOR THE INSANE, GLADESVILLE, NEW SOUTH WALES.

The last report to hand, namely that for 1888, tells us that there were 772 patients in the asylum at the close of the year, being a decrease of six on those of the previous year; but there are still 57 patients in excess of the number for which the asylum was constructed. In spite of this overcrowding the asylum would seem to be carrying out its proper functions, for we learn that 60 per cent. of the admissions were discharged recovered, while the death-rate was only 7·55 on the average number resident. Dr. Manning, the Inspector-General of Asylums for the Colony, says in his official report that he has "to express a general satisfaction with the condition of the hospital, which is, except in some minor respects, in good order."

### STATE HOMŒOPATHIC ASYLUM AT MIDDLETOWN, NEW YORK.

This report runs to nearly one hundred and fifty pages, and is one of the largest we have yet had to notice. The medical superintendent (Dr. Tallcott) has lately paid a long visit to Europe, and he details the various methods of dealing with the insane as regards asylum construction. There is also a long letter from Dr. Peters, of Gheel, setting forth the advantages of a lunatic colony. Dr. Tallcott, however, thinks that the system pursued at Gheel is just as susceptible to improvement as is the American method, and we entirely agree with him; but we do not wholly endorse his statement about the conservatism of Europe preventing improvements, at least his remark is by no means universally applicable. We have a long dissertation—too long for its place, we should say—on the influence of diet in insanity, and a good many paragraphs showing the diet in use in different races and the effects which different kinds of food may be expected to induce. The old story about the Scotchman and the oatmeal is repeated, but not correctly, and Dr. Tallcott sums up as follows: "Now to those who are already, by unwise habits of eating, drinking, and working, the victims of nervousness or nerve exhaustion, it is proper to suggest these important facts: Lean meat stimulates; fat meat relieves nervous erethism; vegetables sustain life in a moderate and healthful manner; fruit cools and purifies the blood, and aids in making the general system pure; grain foods and milk nourish, upbuild, and recuperate the life forces."

There are about 500 patients in the asylum. The admissions during the year reached 217; the discharges 213, and the deaths 36.

There are three medical officers and two "internes," which, we presume, means clinical clerks.

### NORTHAMPTON LUNATIC HOSPITAL, MASSACHUSETTS.

This is a report which may be always read with pleasure and profit. There were 446 patients in the asylum at the close of the year, being a decrease of 35 on the numbers for the previous year. The admissions were 155, the discharges 190, and the deaths 25. General paralysis accounted for six of the deaths and consumption for five.

In many of the American asylums the amusement of the patients plays even a more important part in their treatment than it does in England. Excluding Sundays, there were 243 entertainments during the year in the Northampton Hospital, and these entertainments were of the most varied nature.

When dealing with the question of "relapses," and the difficulties of prognosis in many cases, Dr. Nims says: "The science of medicine is not an exact science. Like physicians in general practice, we can only judge of the probabilities in each case according to our knowledge. The conditions may be obscure or unknown; the causes which may afterwards affect them cannot be taken into account."

There are four medical officers, and one of these is a lady. Probably our cousins might learn something from our system of management, but the appointment of lady physicians for the female departments of our large county asylums is one of the things in which we might well imitate them. It is a wonder that no one of our medical superintendents has yet been bold enough to try the innovation. Perhaps there would be a difficulty in finding suitable candidates; or is it prejudice that stops the way?

### STATE LUNATIC HOSPITAL AT HARRISBURG, PENNSYLVANIA.

At the beginning of the year this asylum contained 665 patients, and at the close the numbers had risen to 734. There were 210 admissions, 141 discharges, and 59 deaths. No application for admission was refused, but we hear the old cry that the hospital is insufficient for the demands on it. Dr. Gerhard thinks that general paralysis is increasing, and each year sees a larger number sent in to his hospital, and this experience in the States is similar to what pertains in many of our English county asylums. When speaking of the idiots, Dr. Gerhard thinks they should be cared for in separate institutions, but he sensibly adds that until this provision is made they should remain in the State Hospital, as all classes of the insane "are the wards of the State, and the State should make liberal provisions for their care."

One suicide occurred, the first for eight years.

There are five physicians in residence, and two of them are ladies.

